# Altered cytokine expression in *Helicobacter pylori* infected patients with bleeding duodenal ulcer

**DOI:** 10.1186/s13104-019-4310-4

**Published:** 2019-05-15

**Authors:** Ljiljana Milic, Aleksandar Karamarkovic, Dusan Popadic, Ana Sijacki, Ilijana Grigorov, Emina Milosevic, Vladica Cuk, Predrag Pesko

**Affiliations:** 1 Surgical Clinic “Nikola Spasić”, Zvezdara University Medical Center, Faculty of Medicine, University Belgrade, Dimitrija Tucovića 161, 11000 Belgrade, Serbia; 2 Institute of Microbiology and Immunology, Faculty of Medicine, University Belgrade, Dr Subotica 1, 11000 Belgrade, Serbia; 3 Clinic for Emergency Surgery, Emergency Center, University Clinical Center of Serbia, Faculty of Medicine, University Belgrade, Visegradska 26, 11000 Belgrade, Serbia; 4 Department of Molecular Biology, Institute for Biological Research, Despota Stefana 142, 11000 Belgrade, Serbia; 5 Clinic for Digestive Surgery, University Clinical Center of Belgrade, Faculty of Medicine, University Belgrade, Koste Todorovica No6, 11000 Belgrade, Serbia

**Keywords:** Bleeding ulcer, Proinflammatory cytokines, *Helicobacter pylori*, Real-time-PCR

## Abstract

**Objective:**

Peptic ulcer disease is a condition in which an important role has infection with *H. pylori*. The most common complication of peptic ulcer is bleeding. The presence of *H. pylori* triggers local and systemic cytokine signaling which may affect processes such as healing, gastric or duodenal rupture, and carcinogenesis. In this study, we examined the concentrations of IL-1β, IL-6, IL-10, TNF, TGF-β and IL-17A in serum by enzyme immunoassay and their mRNA expressions in periulcer biopsies obtained from patients with bleeding peptic ulcer by means of real-time-PCR.

**Results:**

We have shown that pro-inflammatory IL-6 and TNF concentrations in serum were significantly higher in patients who were infected with *H. pylori*, while the concentrations of TGF-β and IL-17A were significantly lower compared to non-infected subjects. IL-17A expression in periulcer mucosa was significantly higher in patients who were infected with *H. pylori*, while the expression of other cytokines, there was no significant difference compared to non-infected controls. Considering higher serum concentrations in non-infected subjects and higher IL-17A expression in mucosal tissue of infected patients, our data support the studies that found IL-17A has protective role in eradication of *H. pylori* infection in infected patients.

**Electronic supplementary material:**

The online version of this article (10.1186/s13104-019-4310-4) contains supplementary material, which is available to authorized users.

## Introduction

Bleeding is one of the most common complications of duodenal ulcer. European data show that 10–50 of every 100,000 hospitalizations are due to ulcer bleeding [1]. Mucosal damage and the duodenal ulcer formation usually occur as a result of interplay between acidopeptic effects of hydrochloric acid and pepsin, treatment with nonsteroidal anti-inflammatory drugs (NSAIDs), and the presence of *H. pylori* infection [2]. *H. pylori* infection of the gastric mucosa leads to sequence of events that trigger inflammatory response, with production of cytokines and the activation of neutrophils [3]. The peptidoglycan within its cell wall is recognized by the cytoplasm sensor Nod 1, which stimulates the expression of numerous cytokines that promote inflammation (IL-1β, IL-6, IL-8, and TNF) but also cytokines that reduce the inflammatory response (IL-4, IL-10) [4, 5]. Protein virulence factors of *H. pylori* increase TGFβ, but the in acute infection decreased levels of this cytokine were found. This is in contrast to upregulated TGFβ levels in chronic infection [6]. Even though TGF-β is immunosuppressive cytokine that induces T regulatory cells, it is also one of the polarizing cytokines for pro-inflammatory Th17 cells [7]. IL-17A, a major cytokine of Th17 population is upregulated in *H. pylori* infection [8]. The net result of the local pro-inflammatory and anti-inflammatory cytokines interplay remains elusive, although it may significantly influence processes such are lesion healing, rupture of the gastric wall and even carcinogenesis. The aim of this study was to investigate the influence of the infection with *H. pylori* on the local inflammatory response measured by the IL-1β, IL-6, IL-10, TNF, TGF-β and IL-17A mRNA expression in patients with bleeding duodenal ulcer, as well as to determine serum concentrations of IL-1β, IL-6, IL-10, TNF, TGF-β and IL-17A in those patients.

## Main text

### Methods

The study was conducted at the Department of Emergency Surgery of Emergency Center, Clinical Center of Serbia in Belgrade. Written informed consent was obtained from each subject, and the study protocol was approved by the Ethical Committee of University Clinical Center of Serbia. The study included consecutive patients taking NSAIDs aged 20 to 70 years with bleeding duodenal ulcers that fulfilled inclusion and exclusion criteria (successive patients’ samples were collected until the number of 30 patients per group was reached). The study group consisted of 30 patients with *H. pylori* infection, while the control group had 30 patients without *H. pylori* infection, as determined by routine urease test and histology. Patients with diabetes, cancer, chronic inflammatory diseases (e.g. rheumatoid arthritis and psoriasis), or autoimmune diseases were excluded from the study. Demographic data are shown in a Table 1. Blood counts for the *H. pylori* positive participants were: red blood cells (RBC) 3.34 ± 1.26 (10^12^ L^−1^), hemoglobin (Hb) 9.43 ± 2.37 (g/dL), hematocrit (Ht) 0.28 ± 0.07 (vol/vol), and platelets (Plt) 209.10 ± 51.52 (10^9^ L^−1^). Blood counts for the *H. pylori* negative participants were RBC 2.70 ± 0.51 (10^12^ L^−1^), Hb 8.04 ± 1.79 (g/dL), Ht 0.22 ± 0.05 (vol/vol), Plt 233.00 ± 71.22 (10^9^ L^−1^). All patients were given esophagoduodenoscopy, and endoscopic finding was described by Forrest classification [9]. This classification is used to assess risk for rebleeding and mortality [10, 11]. Forrest Ia (FIa) finding was not found. In the study group 5 patients (16.6%) FIb was found, 8 patients (26.6%) FIIa was found, 8 patients (26.6%) FIIb was found, 9 patients (30%) FIIc was found. In the control group 9 patients (30%) FIb was found, 12 patients (40%) FIIa was found, 8 patients (26.6%) FIIb was found, 1 patients (3.3%) FIIc was found.

**Table 1:** Demographic data

Variable	HbP+	HbPØ	Total	p
Gender				
Male	26 (86.7%)	28 (93.4%)	54 (90.0%)	NS
Female	4 (13.3%)	2 (6.6%)	6 (10.0%)	
Age (mean ± SD)	50.9 ± 13.2	53.3 ± 11.8	52.1 ± 12.5	NS

#### RNA isolation, reverse transcription and qPCR for determination of cytokine mRNA expression in gastric mucosa

All RNA related procedures were done by independent investigators blinded to the clinical data. Tissue samples obtained by biopsies were homogenized with Teflon micropestle (Eppendorf) in 2 mL tubes (Eppendorf) in 1 mL of TRIZOL^®^ reagent (Invitrogen). Total RNA from all samples was extracted according to the manufacturer’s instructions. Integrity of extracted RNA was verified by electrophoresis on 1.2% agarose gel. Approximately 1 µg of RNA, determined by optical density reading at 260 nm, was used in the reverse transcriptase reaction using M-MuLV RT (Fermentas) with random hexamers (Fermentas) according to the manufacturer’s instructions. Oligonucleotides for human IL-1β, IL-6, IL-10, TNF, TGF-β, IL-17A and glyceraldehyde-3-phosphate dehydrogenase (GAPDH) sequences were designed using Primer Express (ABI) and purchased from Metabion. Primers were designed so that amplicons spanned intron/exon boundaries to minimize amplification of genomic DNA. Sequences, final concentrations and labels of the oligonucleotides were as shown in Additional file 1: Table S1.

To ensure normalization for the amount of starting cDNA each tested gene was amplified simultaneously with GAPDH. All Real-time PCR experiments were performed in 96-well reaction plates (MicroAmp Optical, ABI) in 20 µL volume/well. PCR mixes for determination of IL-1β-β, IL-6, IL-10, TNF, TGF-β and GAPDH contained 12 µL of master mix (10 µL of 2X Maxima SYBR Green qPCR Master, 1 µL of 20× concentrated primers for the respective genes and 1 µL of water) and 8 µL of appropriate sample diluted 1 to 8 in demineralized water. For determination of IL-17 we used 12 µL of master mix (10 µL of 2X Maxima mix-Probe, Fermentas, and 2 µL of oligonucleotide mixture for the IL-17 and GAPDH containing primers and probes) and 8 µL of appropriate sample diluted 1 to 8 in demineralized water. The plates were sealed with optical adhesive film (ABI), briefly centrifuged at high speed and thereafter placed into the thermocycler (Mastercycler ep Realplex^2^, Eppendorf). The thermal cycle conditions were 95 °C for 4 min followed by 40 cycles that were run for 15 s at 95 °C and for 1 min at 60 °C with the melting curve analysis after the last step of amplification. Melting curve analysis step was omitted for the determination of IL-17 and GAPDH expression. For qPCR and melting curve performing and data acquisition Realplex software (Eppendorf) was used.

The levels of expression of IL-1β, IL-6, IL-10, TNF, TGF-β and IL-17A were standardized against GAPDH gene levels as a reference gene. All assays were performed in duplicates. The expression levels of the target genes were expressed in arbitrary units as the ratio of target and reference gene (1/2^−ΔCt^).

#### Enzyme immunoassays for determination of blood cytokines concentrations

At the enrollment, serum (or plasma for determination of TGF-β) was obtained from a sample of venous blood drawn before endoscopy, and frozen at − 20 °C for later determination of cytokines. The concentrations of IL-1β, IL-6, IL-10, TNF, TGF-β and IL-17A are measured by ELISA tests (eBioscience, ELISA Ready-SET-Go!) Each measurement was done according to the manufacturer’s instructions. The cytokine detection limits in serum were: 1.95 pg/mL for IL-1β; 3.91 pg/mL for IL-6; 0.59 pg/mL for IL-10; 0.975 pg/mL for TNF; 31.25 pg/mL for TGF-β, and 7.81 pg/mL for IL-17A.

#### Statistical analysis

Scale data for mRNA and protein levels of the aforementioned cytokines was tested for normality by Kolmogorov–Smirnov test. Normally distributed data were compared by Student’s t-test. Otherwise, Mann–Whitney U test was applied. Categorical data were compared by Pearson’s Chi Square or Fisher’s exact significance test, where necessary. The p < 0.05 was considered significant. For the statistical analysis SPSS software 11.5 (SPSS Inc., Chicago, IL) was used.

### Results

#### Cytokine mRNA expression in periulcer mucosa

**Fig. 1:**
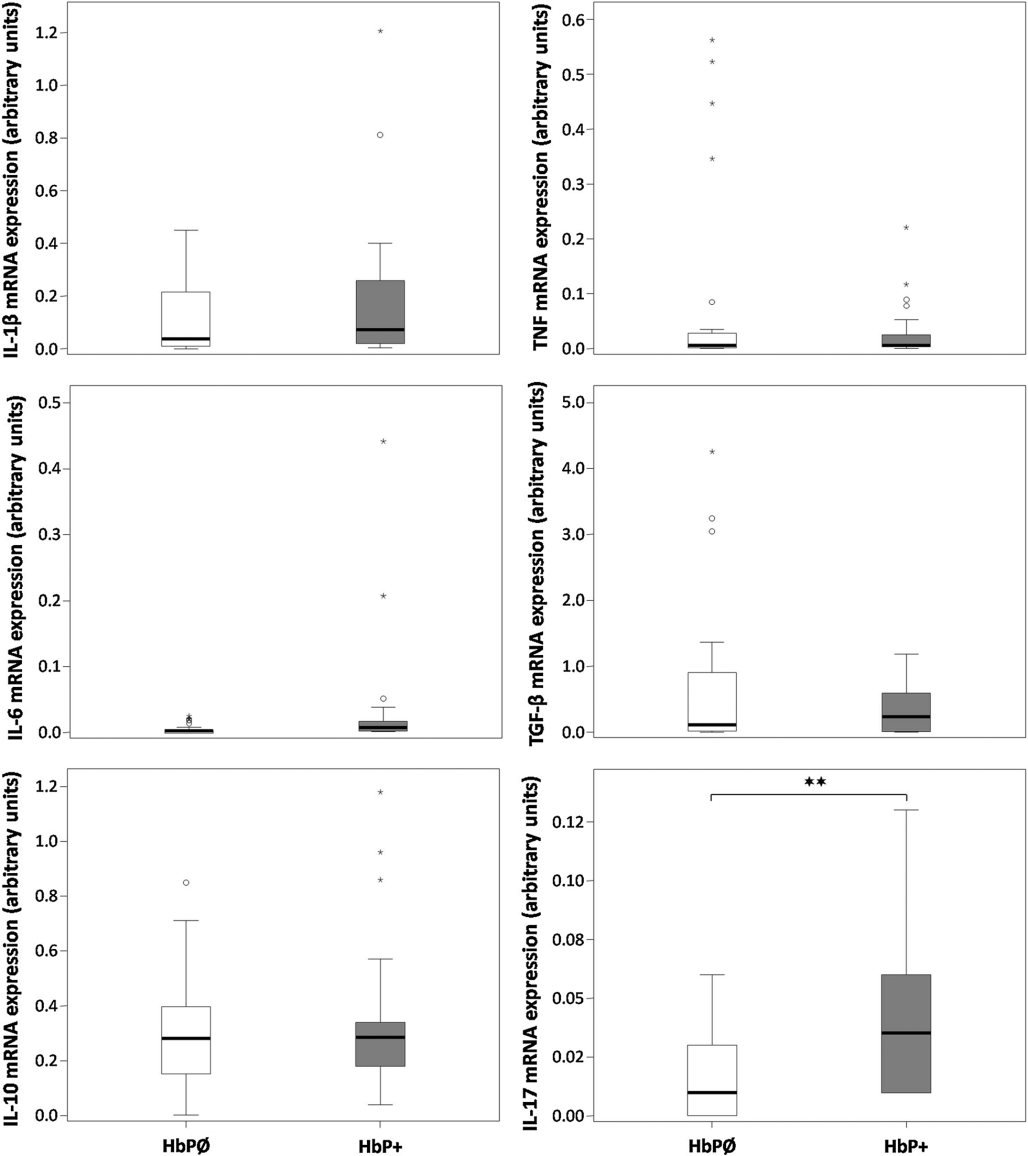
Expression of IL-1β, IL-6, IL-10, TNF, TGF-β i IL-17A mRNAs in gastric mucosa of *H. pylori*-positive patients (HbP+) and the ones without *H. pylori* infection (HbPØ) were quantified by real-time PCR. The relative expression of each target gene is presented in arbitrary units after normalization with GAPDH mRNA expression (see Materials and methods). Data are presented in the box-plot format in which cross line represents median, rectangle represents interquartile range, whiskers represents maximal and minimal values in 1.5 interquartile range, whereas outlier and extreme values are represented by circles and stars respectively, ✶✶p < 0.01

We analyzed IL-1β, IL-6, IL-10, TNF, TGF-β and IL-17A gene expression in periulcer mucosa of *H. pylori* positive and *H. pylori* negative patients by means of qPCR to determine local cytokine response to *H. pylori* infection. The results demonstrated that the expression of IL-17A mRNA of *H. pylori* positive patients was higher in comparison with that of *H. pylori* negative patients (p < 0.01) (Fig. 1). Unexpectedly, the expression of pro-inflammatory mediator TNF mRNA was not significantly increased in mucosa of *H. pylori* positive patients when compared to the values obtained in samples from *H. pylori* negative patients (Fig. 1). There was also no significant difference in expression of anti-inflammatory TGF-β and IL-10 mRNAs between *H. pylori* positive patients and *H. pylori* negative patients (Fig. 1).

#### Blood cytokine concentrations

**Fig. 2:**
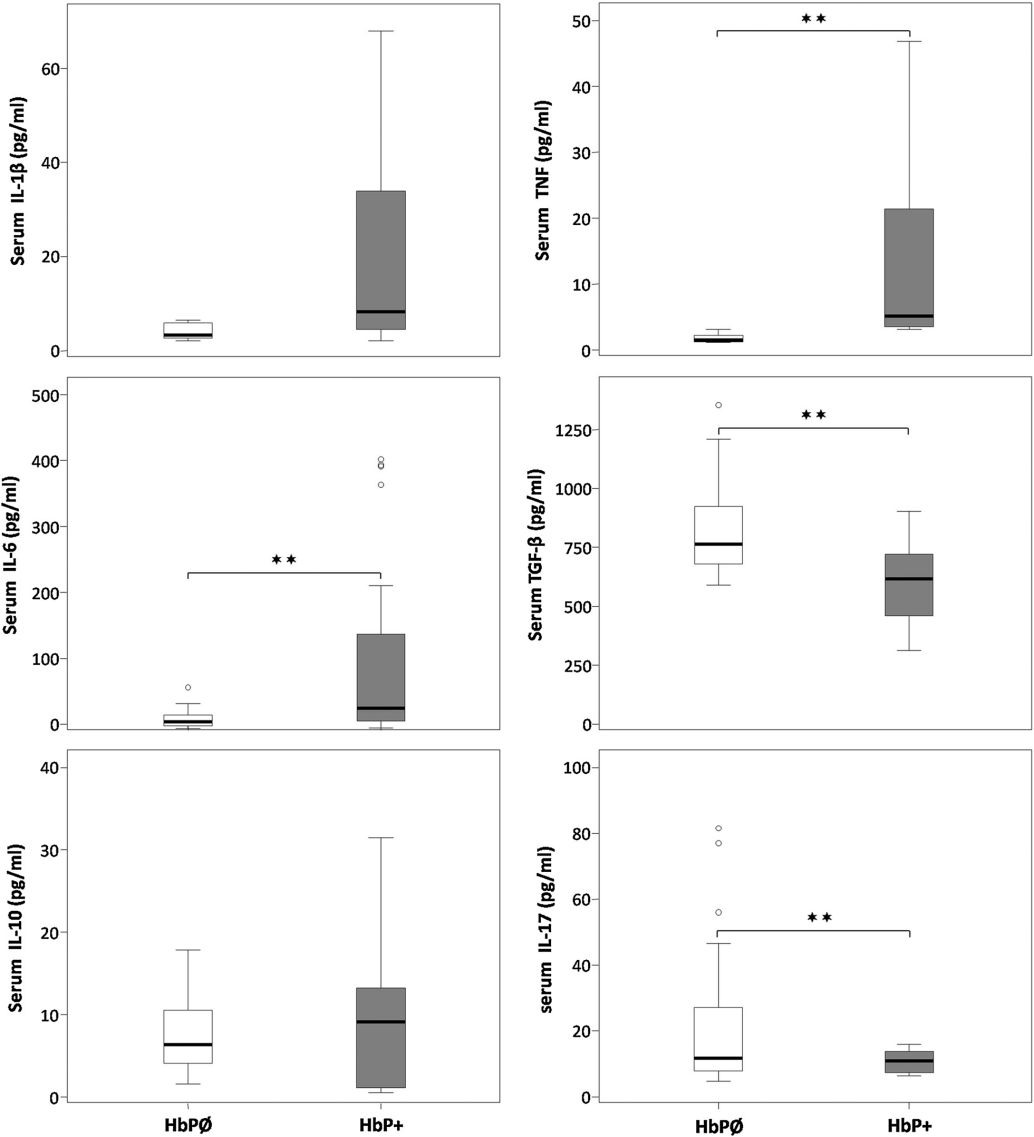
Concentrations IL-1β, IL-6, IL-10, TNF, TGF-β i IL-17A in blood of patients with *H. pylori* infection (HbP+) and without *H. pylori* infection (HbPØ). Data are presented in the box-plot format in which cross line represents median, rectangle represents interquartile range, whiskers represents maximal and minimal values in 1.5 interquartile range, whereas outlier values are represented by circles. ✶p < 0.05, ✶✶p < 0.01

To determine systemic response to *H. pylori* infection we measured concentrations IL-1β, IL-6, IL-10, TNF, TGF-β and IL-17A in blood samples of all patients (Fig. 2). The concentrations of TGF-β and IL-17A were significantly higher in non-infected patients (Fig. 2) than in patients with *H. pylori* infection. Concentrations of IL-1β, IL-6, IL-10, and TNF inclined toward higher values in patients with *H. pylori* infection compared to those without infection but the difference did not reach the level of statistical significance. Cytokine detectability was: TGF-β—100%, IL-1β—35.0%, IL-6—85.0%, IL-10—56.7%, TNF—28.3%, and for IL-17—50.0%. Proportion of cytokine detectable samples did not differ statistically significantly between *H. pylori* positive and negative patients, except for IL-17, where the proportion of positive samples was significantly higher in the *H. pylori* negative patients.

### Discussion

The analysis of IL-1β mRNA expression in biopsy material, demonstrated that most patients had detectable values of IL-1β mRNA. The difference in IL-1β mRNA between these two groups was not significantly significant but the median of IL-1β mRNA expression was higher in patients with *H. pylori* infection. Similar data was published by several groups, and it is generally accepted that *H. pylori* infection increases of IL-1β mRNA expression as a local immune response [3, 12, 13]. Expectedly, median values of serum IL-1β were higher in the group with *H. pylori* infection, but without statistical significance.

Measurements of circulating IL-1β revealed that only a third of patients had value within the limits of sensitivity of the test. Since all the patients were using NSAIDs, one can speculate that these drugs hindered several cytokines’ production to undetectable levels in a portion of patients. NSAIDs used by our patients were diclofenac and ibuprofen. Ibuprofen did not affect plasma cytokine levels (namely IL-1β, TNF, IL-6, and IL-10) of healthy people [14]. However, there is conflicting data on effect of ibuprofen on cytokine levels in specific tissues [15, 16]. Diclofenac reduced plasma TNF in a study on mildly obese volunteers [17] so it could be that it affected detectability of this cytokine in our hands, too. Yet in the same study, diclofenac did not altered IL-10 levels, and IL-1β and IL-6 were omitted from the analysis due to high rate of undetected levels, even though the more sensitive platform (bead-based multiplex sandwich immunofluorescence assay vs. ELISA) was used. To the best of our knowledge, there is no data how ibuprofen and diclofenac affect serum IL-17A levels.

Literature search did not reveal any article investigating IL-1β in sera of patients with bleeding duodenal ulcer in relation to *H. pylori* infection.

Local IL-6 mRNA expression was significantly higher in *H. pylori* infected patients compared to non-infected, resembling to the results reported by Ströberg et al. [3]. Another study, carried out by Ren et al. has shown elevated IL-6 in antral biopsy of patients with chronic gastritis when infected with *H. pylori*, measured by enzyme immunoassay [18]. Expectedly, serum IL-6 values were significantly higher in patients with *H. pylori* infection compared to non-infected. The results of the IL-6 serum concentrations in our study are in line with those from Cichoz-Lach et al. who demonstrated that *H. pylori* infected, but not non-infected patients with erosive gastritis have higher values of IL-6 compared to matched healthy controls [19].

The difference in TNF mRNA expression between the two groups of patients in duodenal biopsy material did not reach the level of statistical significance. Bontems et al. also found very similar values of TNF between patients with dyspepsia infected and uninfected with *H. pylori* [20]. Our finding partially disagrees with the previously reported data from Goll et al. demonstrating generally higher TNF mRNA expression in mucosa following *H. pylori* infection [4]. However, TNF mRNA expression in aforementioned study was lowest in patients with duodenal ulcers compared to other *H. pylori* infected patients, approaching values determined in non-infected patients and without statistical significance. TNF in serum was increased in infected patients, presumably as a response to the infection. However, further studies addressing serum TNF in *H. pylori* infected patients are warranted, since equivocal results were published previously, probably due to different clinical settings [21–23].

Our study demonstrates that half of patients without *H. pylori* infection had a detectable IL-17A mRNA in the biopsy tissue, while this cytokine mRNA was measurable in all the infected patients. Patients infected with *H. pylori* had significantly higher IL-17A mRNA expression in periulcer mucosa than non-infected patients. These results were expected due to the pro-inflammatory effect of infection with *H. pylori* and the type of the inflammatory response driven by IL-17A producing T cells e.g. recruitment of neutrophils. Our results are in the concordance with previously reported data [24]. Unexpectedly, serum concentrations of IL-17A were higher in patients without *H. pylori* infection. There is no published data to compare such a finding. Interestingly, higher serum concentration of IL-17 in uninfected patients might be protective in combating *H. pylori* [25, 26]. Jafarzadeh and coworkers reported higher serum levels of IL-17A in duodenal ulcer patients compared to healthy subjects and asymptomatic *H. pylori* infected individuals [27], but our result is based on the different study setup and therefore warrants replication.

IL-10 mRNA expression levels did not differ significantly in the two groups of our patients. In a study from Bontems et al. performed in 45 adults and children in Belgium, IL-10 mRNA expression was higher in infected children compared to non-infected, while in adults there was no differences between groups regarding *H. pylori* status [20]. In the study from Norway by Goll et al. [4] that included 91 subjects, IL-10 mRNA expression was elevated in mucosa of *H. pylori* positive patients compared to non-infected ones. This inconclusiveness could be partially explained by the heterogeneity of the patients groups. Patients recruited in our study had acute ulcer bleeding and took NSAIDs that may considerably affect local IL-10 mRNA expression by interfering with eicosanoids synthesis. Serum concentrations of IL-10 in serum, were similar in both groups of our patients resembling to the study of Russo and coworkers [22].

Although there was no statistically significant difference in TGF-β mRNA expression between compared groups of patients, the median value of TGF-β was higher in patients with *H. pylori* infection, consistently with the previously reported data [3, 28, 29]. TGF-β in plasma was decreased in *H. pylori* infected patients compared to non-infected in line with results reported by Ki et al. demonstrating inverse relation between TGF-β and anti *H. pylori* antibodies in blood [30].

We found higher serum concentrations in non-infected subjects and higher IL-17A expression in mucosal tissue of infected patients. Our data indicate that IL-17A might have protective role in *H. pylori* infection eradication in patients with bleeding ulcers. However, further studies assessing in-depth cellular and molecular mechanism of defense in *H. pylori* infection in larger cohorts of patients are necessary to address weather IL-17A has this protective role.

## Limitations

A small number of patients were examined in the study, so a study involving a larger number of participants was required to confirm the results. Small sample size might be the reason of higher proportion of men with bleeding ulcer in this study compared to other studies in Serbian population [31].

## Additional file

**Additional file 1: Table S1.** Sequences, final concentrations and labels of the oligonucleotides.

## Data Availability

The datasets generated and/or analyzed during the current study are not publicly available due to the patients’ privacy policy but are available from the corresponding author on reasonable request.

## Abbreviations

HbP: *Helicobacter pylori*
TNF: tumor necrosis factor
IL: interleukin
TGF-beta: transforming growth factor-beta
Real-time-PCR: real-time polymerase chain reaction
