# Supplementary material for: Altered cytokine expression in Helicobacter pylori infected patients with bleeding duodenal ulcer
Source: BMC Res Notes. 2019 May 15;12:278. doi: 10.1186/s13104-019-4310-4 (PMC6521506; doi:10.1186/s13104-019-4310-4)
Supplement: Supplementary file 1 — Additional file 1: Table S1. Sequences, final concentrations and labels of the oligonucleotides. [file 13104_2019_4310_MOESM1_ESM.docx]

Table S1. Sequences, final concentrations and labels of the oligonucleotides

| Oligo | Target primer/probe sequence (5′-3′) | Concentration (nM) | Labels | Sequence and position |
| --- | --- | --- | --- | --- |
| IL-1β |  |  |  | NM_000576 |
| F | GCTGCTCTGGGATTCTCTTCAG | 300 | NA | 33 |
| R | TGGCGAGCTCAGGTACTTCTG | 300 | NA | 112 |
|  |  |  |  |  |
| IL-6 |  |  |  | NM_000600 |
| F | CCAGGAGCCCAGCTATGAACT | 300 | NA | 103 |
| R | AGCAGCCCCAGGGAGAAG | 300 | NA | 172 |
|  |  |  |  |  |
| IL-10 |  |  |  | NM_000572 |
| F | GGGAGAACCTGAAGACCCTCA | 100 | NA | 397 |
| R | TGCTCTTGTTTTCACAGGGAAG | 300 | NA | 468 |
|  |  |  |  |  |
| TNF |  |  |  | NM_000594 |
| F | GCCCAGGCAGTCAGATCATC | 100 | NA | 395 |
| R | AGCTGCCCCTCAGCTTGA | 300 | NA | 480 |
|  |  |  |  |  |
| TGF β |  |  |  | NM_000660 |
| F | CTGCTGAGGCTCAAGTTAAAAGTG | 300 | NA | 1351 |
| R | TGAGGTATCGCCAGGAATTGT | 300 | NA | 1429 |
|  |  |  |  |  |
| IL-17 |  |  |  | NM_002190 |
| F | CATCCATCCCCAGTTGATTGG | 100 | NA | 14 |
| Taqman | AAGACCTCATTGGTGTCACTGCTACTGCTG | 200 | 5'FAM-TAMRA3' | 58 |
| R | GATTTCGTGGGATTGTGATTC | 100 | NA | 136 |
|  |  |  |  |  |
| GAPDH |  |  |  | NM_002046 |
| F | CATCCATGACAACTTTGGTATCG | 300 | NA | 591 |
| Taqman | AAGGACTCATGACCACAGTCCATGCC | 200 | 5'VIC-TAMRA3' | 617 |
| R | CCATCACGCCACAGTTTCC | 300 | NA | 698 |
